# The Relationship Between Cardiac Troponin in People Hospitalised for Exacerbation of COPD and Major Adverse Cardiac Events (MACE) and COPD Readmissions

**DOI:** 10.2147/COPD.S432166

**Published:** 2023-11-06

**Authors:** Constantinos Kallis, Amit Kaura, Nathan A Samuel, Abdulrahim Mulla, Ben Glampson, Kevin O’Gallagher, Jim Davies, Dimitri Papadimitriou, Kerrie J Woods, Anoop D Shah, Bryan Williams, Folkert W Asselbergs, Erik K Mayer, Richard W Lee, Christopher Herbert, Stuart W Grant, Nick Curzen, Iain B Squire, Thomas Johnson, Ajay M Shah, Divaka Perera, Rajesh K Kharbanda, Riyaz S Patel, Keith M Channon, Jamil Mayet, Jennifer K Quint

**Affiliations:** 1 National Heart and Lung Institute, Imperial College London, London, UK; 2 School of Public Health, Imperial College London, London, UK; 3 NIHR Imperial Biomedical Research Centre, Imperial College London and Imperial College Healthcare NHS Trust, London, UK; 4 NIHR Oxford Biomedical Research Centre, University of Oxford and Oxford University Hospitals NHS Foundation Trust, Oxford, UK; 5 NIHR King’s Biomedical Research Centre, King’s College London and King’s College Hospital NHS Foundation Trust, London, UK; 6 NIHR University College London Biomedical Research Centre, University College London and University College London Hospitals NHS Foundation Trust, London, UK; 7 Institute of Health Informatics, University College London, London, UK; 8 Imperial Clinical Analytics, Research & Evaluation (iCARE) and Department of Surgery & Cancer, Imperial College London, London, UK; 9 Early Diagnosis and Detection Centre, NIHR BRC at The Royal Marsden and Institute of Cancer Research, London, UK; 10 NIHR Leeds Clinical Research Facility, Leeds Teaching Hospitals Trust and University of Leeds, Leeds, UK; 11 NIHR Manchester Biomedical Research Centre, Manchester University NHS Foundation Trust and the University of Manchester, Manchester, UK; 12 NIHR Southampton Clinical Research Facility and Biomedical Research Centre, Faculty of Medicine, University of Southampton and University Hospital Southampton NHS Foundation Trust, Southampton, UK; 13 NIHR Leicester Biomedical Research Centre, Glenfield Hospital, and Department of Cardiovascular Sciences, University of Leicester, Leicester, UK; 14 NIHR Bristol Biomedical Research Centre, University of Bristol and University Hospitals Bristol NHS Foundation Trust, Bristol, UK; 15 NIHR Guys & St Thomas’ Hospital Clinical Research Facility, King’s College Hospital, and King’s College London British Heart Foundation Centre of Excellence, London, UK

**Keywords:** COPD, CVD, exacerbation

## Abstract

**Background:**

No single biomarker currently risk stratifies chronic obstructive pulmonary disease (COPD) patients at the time of an exacerbation, though previous studies have suggested that patients with elevated troponin at exacerbation have worse outcomes. This study evaluated the relationship between peak cardiac troponin and subsequent major adverse cardiac events (MACE) including all-cause mortality and COPD hospital readmission, among patients admitted with COPD exacerbation.

**Methods:**

Data from five cross-regional hospitals in England were analysed using the National Institute of Health Research Health Informatics Collaborative (NIHR-HIC) acute coronary syndrome database (2008–2017). People hospitalised with a COPD exacerbation were included, and peak troponin levels were standardised relative to the 99th percentile (upper limit of normal). We used Cox Proportional Hazard models adjusting for age, sex, laboratory results and clinical risk factors, and implemented logarithmic transformation (base-10 logarithm). The primary outcome was risk of MACE within 90 days from peak troponin measurement. Secondary outcome was risk of COPD readmission within 90 days from peak troponin measurement.

**Results:**

There were 2487 patients included. Of these, 377 (15.2%) patients had a MACE event and 203 (8.2%) were readmitted within 90 days from peak troponin measurement. A total of 1107 (44.5%) patients had an elevated troponin level. Of 1107 patients with elevated troponin at exacerbation, 256 (22.8%) had a MACE event and 101 (9.0%) a COPD readmission within 90 days from peak troponin measurement. Patients with troponin above the upper limit of normal had a higher risk of MACE (adjusted HR 2.20, 95% CI 1.75–2.77) and COPD hospital readmission (adjusted HR 1.37, 95% CI 1.02–1.83) when compared with patients without elevated troponin.

**Conclusion:**

An elevated troponin level at the time of COPD exacerbation may be a useful tool for predicting MACE in COPD patients. The relationship between degree of troponin elevation and risk of future events is complex and requires further investigation.

## Introduction

Comorbidities are common in people with COPD. Specifically, ischaemic heart disease (IHD) and cardiac arrhythmias are the most prevalent comorbidities, and account for a significant proportion of deaths.^1^,^2^ Further, acute exacerbations of COPD are associated with increased mortality, and over 20% of people with COPD have at least one moderate (managed in primary care) or severe (hospitalised) exacerbation per year.^3^ COPD exacerbation is a presumptive diagnosis based upon deterioration of symptoms. However, it may be difficult to accurately identify alternative diagnoses due completely or partially to comorbid conditions if there is overlap of the symptoms. This can, in turn, impact on subsequent management and prognosis.

Furthermore, people with COPD have an increased risk of acute CV events around the time of an exacerbation.^4^ Whilst troponin is almost universally measured in patients presenting to hospital with symptoms of acute COPD exacerbation, interpretation of an elevated high sensitivity troponin (hsTrop) is challenging in this context, given the possibility that the result may reflect myocardial injury, Type 2 or Type 1 MI.^5^ The clinical implications of an elevated hsTrop are therefore currently unclear, especially given that 20% of hospital inpatients have hsTrop levels above the upper limit of normal.^6^ Previous studies have suggested that exacerbations are associated with elevated troponin levels and that COPD patients with elevated troponin levels of ≥0.04 μg·L^−1^ at exacerbation are at increased risk of death post hospital discharge.^7^,^8^ However, studies have also suggested that peak troponin levels post-MI, in people with COPD, are not as high as in those without, suggesting that the interpretation of troponin levels in people with COPD may not be straightforward.^9^

Together with typical symptoms, elevation of troponin assays now form an important component for diagnosis of acute coronary syndrome, often in conjunction with ECG abnormalities.^9^ Specifically, dynamic elevation of troponin exceeding the manufacturer’s 99th percentile (considered as the upper limit of normal (ULN) in routine practice) is typically diagnostic of a MI.^10^ However, such values will be seen in 20% of all hospital patients, including both inpatients and outpatients, due most frequently to either myocardial injury or Type 2, as opposed to Type 1, MI, and the degree of elevation is associated with subsequent medium-term mortality, regardless.^11^ Importantly, any detectable troponin, even when below the 99th centile level, is still associated with adverse clinical outcomes.^12^ Few studies have investigated mortality risk in a secondary care population of exacerbating COPD patients, stratified by troponin levels, and there is no detailed analysis of data exploring the subsequent management of these patients. Therefore, our hypothesis was that hsTrop may be associated with clinical outcomes in patients admitted to hospital with an acute exacerbation of COPD. Consequently, we aimed to investigate the relationship between hsTrop and (1) major adverse cardiac events (MACE) including all-cause mortality and (2) COPD hospital readmission.

## Methods

### Database

This study was conducted using data collected from the electronic National Institute of Health Research Health Informatics Collaborative (NIHR-HIC) acute coronary syndrome database. This database contained information on 257,948 patients across five hospitals (Imperial College Healthcare, Guys and St Thomas’ Hospital, Kings College Hospital, Oxford University Hospital, University College Hospital), starting from 2010 (2008 for University College Hospital) until 1 April 2017. 156,410 (60.6%) patients had an inpatient troponin assay recorded. The dataset contained detailed information on patient demographics, diagnoses at admission, biochemistry/ haematological results, echocardiography and mortality. Diagnoses were defined from the 10th revision of the International Statistical Classification of Diseases and Related Health Problems (ICD-10) discharge codes. 134,517 patients (86% of recorded troponin) had ICD-10 discharge codes recorded.

### Study Population

**Figure 1:**
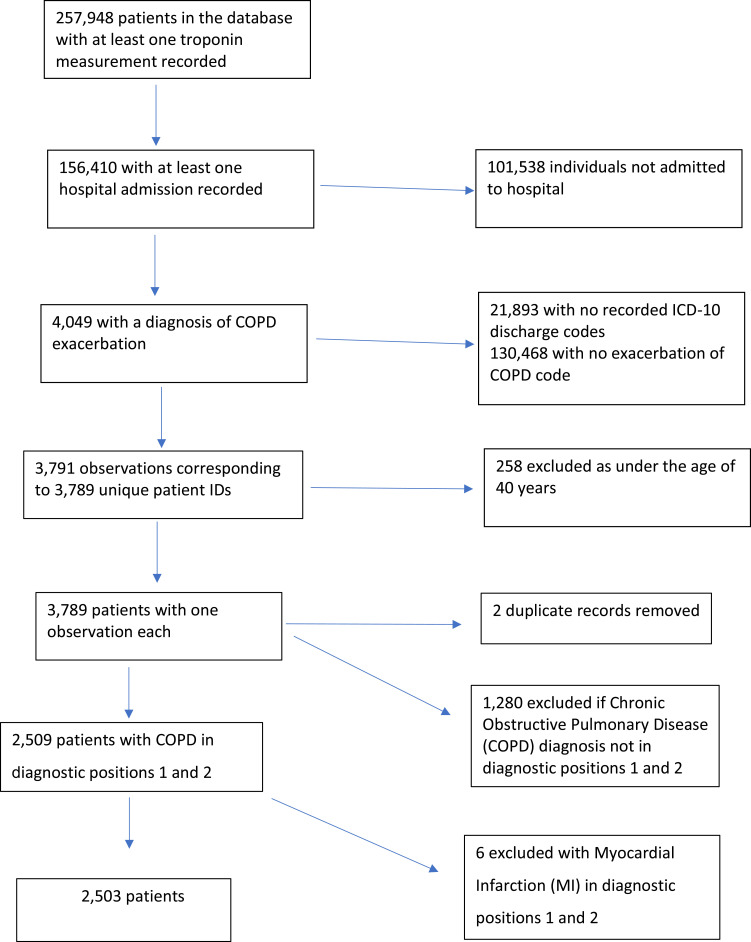
Study diagram of participant inclusion.

The inclusion criteria were patients with a diagnosis of a COPD exacerbation at initial admission (Figure 1). An exacerbation was defined by a primary diagnosis indicated by one of three ICD-10 codes; J44.0, J44.1, J44.9 or the use of J44.0 or J44.1 in a secondary diagnostic position with a lower respiratory tract infection code (J22) in position 1. This classification was shown to have an 87.5% sensitivity and 50.2% positive predictive value when referenced to Hospital Episodes Statistics data (HES) for identifying COPD exacerbations compared with other code combinations.^12^ Patients who experienced a diagnosed acute coronary syndrome (ACS) during that admission were excluded as this study aimed to investigate the prognostic significance of troponin in a population of people admitted to hospital with a COPD exacerbation.

### Co-Variables and Outcomes

The primary outcome was time to MACE within 90 days from peak troponin measurement. MACE outcome was defined as non-fatal myocardial infarction (excluding old myocardial infarction), admission with heart failure or stroke (defined as diagnostic positions 1 and 2 after troponin measurement during the same time period), coronary revascularisation (defined as having PCI (Percutaneous Coronary Intervention) or CABG (Coronary Artery Bypass Grafting)) or having died from any cause within 90 days from peak troponin measurement. Time to COPD hospital readmission within 90 days was also investigated as secondary outcome.

Variables were included based on previous clinical knowledge and included demographic and clinical risk factors for cardiovascular disease. Demographic factors were age and gender. Clinical risk factors included the following comorbidities: hypertension, previous myocardial infarction, hypercholesterolaemia, heart failure, peripheral arterial disease (PAD), malignancy, chronic kidney disease (CKD) and diabetes mellitus (DM). Other variables included haematological and biochemistry results e.g., C-reactive protein (CRP), haemoglobin, white cell count, creatinine and platelet counts. Comorbidities and risk factors were defined based on combinations of keywords that had been developed during curation of the database to correspond to ICD-10 code definitions as per previous methodology.^13^

### Statistical Analysis

Peak hsTrop measurements (the highest troponin level recorded during the initial admission) were standardised to allow for differing troponin assays. Standardisation was calculated by dividing the troponin result by the ULN, giving a ratio relative to the ULN. If only one troponin assay was recorded, this was used as the peak troponin. A standardised troponin value that was ≥1 typically described a clinically significant elevation as it indicated a troponin level above the ULN.^10^

#### Baseline Characteristics and Elevated Troponin

Descriptive analyses were summarised as median (interquartile range; IQR) for continuous variables and number (percentage) for categorical variables. Mann–Whitney tests were implemented to compare medians between patients with and without elevated troponin and Chi-square tests were used to compare proportions between the same groups of patients.

#### Time to Event Analysis Within 90 Days Using Troponin as Binary Exposure (Elevated vs Not)

We compared time to MACE within 90 days and time to COPD readmission within 90 days from peak troponin measurement between patients with elevated troponin level and patients with non-elevated levels. Cox proportional hazard regression modelling was utilised to calculate hazard ratios (HRs) and 95% confidence interval (CI) for these comparisons. Three model specifications were implemented for these comparisons: unadjusted, adjusted only for demographic variables (age and gender) and fully adjusted (demographic variables, other co-morbidities in addition to haematological and biochemistry results).

#### Time to Event Analysis Within 90 Days of Log-Transformed Troponin Level as a Continuous Exposure

We log-transformed standardised troponin using base-10 logarithm to consider troponin distribution skewness and to evaluate the association of each 10-fold increase of standardised troponin.^14^ We then explored the relationship between peak troponin value and time to MACE within 90 days implementing a logarithmic transformation (base-10 logarithm) (log_10_ troponin) using statistically significant polynomial terms up to fourth degree.^14^

Where relationships were non-linear (i.e. based on polynomial terms), we have included graphical representations of the relationship and the corresponding tables with estimates for representative troponin values based on Stata commands margins and marginsplot. These visual aids are only utilised to make complex non-linear model-based effects more tangible by computing predicted or expected values for hypothetical or prototypical cases.^15^ These methods are not suitable to determine the statistical significance of the relationship between troponin and each outcome of interest as each estimate is based on a relatively small number of observations.

We estimated the overall predictive performance of this model using Harrell’s C and Gonen and Heller’s K concordance coefficients. In addition, we evaluated the overall predictive performance of Cox regression models that included significant standardised troponin polynomial terms for patients with elevated troponin.

#### Prediction of MACE Within 1 Year

We investigated the relationship between peak standardised troponin and having at least one MACE event within a year using logistic regression. As with the Cox models, we implemented logarithmic transformation (base-10 logarithm) in our analysis (log_10_ troponin) using statistically significant polynomial terms up to fourth degree. We also evaluated the overall predictive performance of this model using Area under ROC curve (AUC). As our sample size was relatively small, we estimated Leave-One-Out-Cross-Validation (LOOCV) AUC that has been shown to be a suitable resampling technique for this sample size magnitude.^16^

Statistical analyses were carried out using R-Studio software (version 4.0.0) and Stata/MP 17.0 with statistical significance set to a two-tailed p-value <0.05.

### Sensitivity Analysis

Given troponin elevations can be caused by other clinical events, e.g. pulmonary embolism, heart failure, myocarditis, myocardial contusion and sepsis, we repeated the analysis excluding individuals who had any of those conditions in the secondary diagnostic position.

## Results

### Baseline Characteristics and Elevated Troponin

There were 2487 patients included in our analysis.^12^ As only 6 patients were diagnosed with an ACS during the COPD exacerbation admission they were excluded (Figure 1). 377 (15.2%) patients had a MACE event within 90 days from peak troponin measurement. Of these, 336 (89.1%) were mortality events. 203 (8.2%) were readmitted within 90 days from peak troponin measurement. 1107 (44.5%) had an elevated troponin level. The median patient age was 75 years (IQR: 67–82 years) for patients without MACE and 78 years (IQR: 71–85 years) for those with MACE. There was no difference in the proportion of men and women with MACE. CRP, white cell count and creatinine were significantly higher for patients with MACE and heart failure, malignancy and chronic kidney disease (CKD) were more prevalent in patients with MACE compared with patients without MACE. Out of 2487 patients, 43 (1.7%) had PCI at least a day after they had their peak troponin measurement. An additional 21 patients (0.8%) had angiography without PCI. Within 90 days, only 14 patients (0.6%) had PCI. An additional 11 patients (0.4%) had angiography without PCI. The baseline characteristics for the study cohort are summarised in Table 1.

**Table 1:** Baseline Characteristics for Patients Without and With MACE Events in 90 Days

Variable	Without MACE (n=2110)	With MACE (n=377)	p-value
**Demographic**			
Age (years)	75 (67–82)	78 (71–85)	<0.001
Female	966 (45.8)	158 (41.9)	0.164
**Laboratory results**			
Haemoglobin (g/dL)	13.5 (12.2–14.7)	12.4 (10.9–14.1)	<0.001
CRP (mg/L)	27 (8–83)	45.1 (16.1–118.8)	<0.001
White cell count (x10^9^/L)	10.8 (8.2–14.1)	11.2 (8.2–15.8)	0.031
Creatinine (mmol/L)	75 (63–97)	81 (65–118)	<0.001
Platelet (x10^9^/L)	246 (195–307)	253 (187–334)	0.177
**Risk factors**			
Hypertension	605 (28.7)	101 (26.8)	0.455
Previous MI	76 (3.6)	17 (4.5)	0.392
Hypercholesterolaemia	299 (14.2)	40 (10.6)	0.063
Heart Failure	218 (10.3)	78 (20.7)	<0.001
Peripheral artery disease	65 (3.1)	16 (4.2)	0.241
Malignancy	188 (8.9)	69 (18.3)	<0.001
Chronic Kidney Disease	84 (4.0)	29 (7.7)	0.001
Diabetes	349 (16.5)	58 (15.4)	0.576

Out of 1107 patients with an elevated troponin level, 256 (23.1%) had a MACE event and 101 (9.1%) had a COPD readmission within 90 days from peak troponin measurement. For 1380 patients with a non-elevated troponin measurement, the corresponding numbers of patients were 121 (8.8%) and 102 (7.4%), respectively. Using a Chi-square test, we found that the percentage of patients with elevated troponin who had a MACE event within 90 days from peak troponin measurement (23.1%) was significantly higher compared with the corresponding percentage for patients with non-elevated troponin (8.8%) (p<0.001).

#### Time to Event Analysis Within 90 Days Using Troponin as Binary Exposure (Elevated vs Not)

Patients with elevated troponin had a higher risk of MACE when compared with patients without elevated troponin; unadjusted HR 2.88 (95% CI 2.32–3.58). Adjusted for demographic variables HR 2.64 (95% CI 2.12–3.30) and full adjustment HR 2.20 (95% CI 1.75–2.77). COPD patients with elevated troponin levels experienced COPD readmissions quicker when compared with patients without elevated troponin; unadjusted HR 1.39 (95% CI 1.06–1.83). This was attenuated on adjusting for confounders; fully adjusted HR 1.37 (95% CI 1.02–1.83).

We implemented sensitivity analysis by excluding patients with at least one of the following diagnoses in any secondary position at baseline: pulmonary embolism, heart failure, myocarditis, myocardial contusion and sepsis. Out of 2487 patients, 265 (10.7%) were excluded from our analysis. Our sensitivity analysis sample included 2222 patients.

In this sample, patients with elevated troponin had a higher risk of MACE when compared with patients without elevated troponin. The unadjusted HR was 3.07 (95% CI 2.41–3.90). Adjusting for demographic variables, the adjusted HR was 2.84 (95% CI 2.23–3.63). The full adjustment HR was 2.43 (95% CI 1.89–3.13). COPD patients with elevated troponin were more likely to experience COPD readmissions quicker when compared with patients without elevated troponin. The unadjusted HR was 1.47 (95% CI 1.10–1.98). The corresponding fully adjusted HR was 1.47 (95% CI 1.08–2.00).

#### Time to Event Analysis Within 90 Days of Log-Transformed Troponin Level as a Continuous Exposure

**Figure 2:**
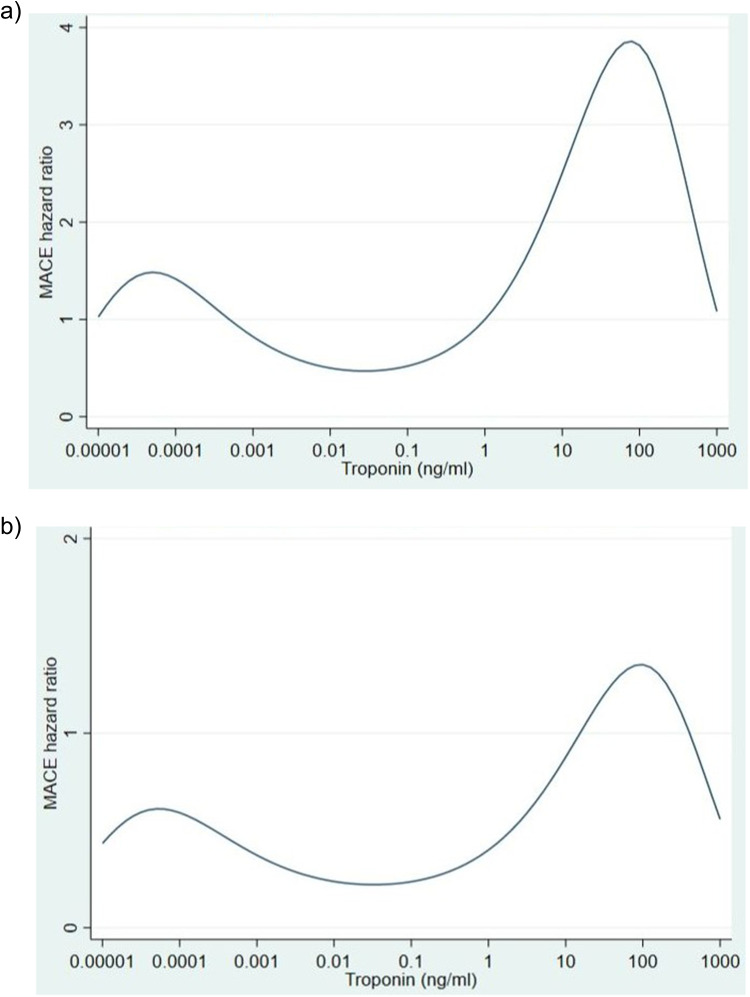
(**a**) Predicted unadjusted hazard ratio for MACE events (**b**) Predicted adjusted hazard ratio for MACE events.

For each 10-fold increase of troponin, the effect increased and then decreased twice, below and above peak troponin threshold (Table 2 and Figure 2a and b). This table and figures should be used only as visual aids of the complex non-linear relationship between troponin and time to MACE events. Estimates at each troponin value should not be used to determine the statistical significance of the effect as they are based on a relatively small number of observations.

**Table 2:** Unadjusted and Adjusted Hazard Ratio Estimates for MACE Events

Troponin (ng/mL)	Unadjusted HR	Adjusted HR
0.00001	1.03	1.03
0.0001	1.42	1.42
0.001	0.82	0.82
0.01	0.50	0.50
0.1	0.52	0.52
1	N/A	N/A
10	2.51	2.51
100	3.82	3.82
1000	1.08	1.08

Harrell’s C coefficient (the default option using Stata estat concordance command) was estimated to be equal to 0.73. Gonen and Heller’s K coefficient was not sensitive to the degree of censoring as Harrell’s C coefficient and it was slightly lower (0.68). Both coefficients were comparable to the Area under the ROC curve (AUC) statistic for binary logistic regression models.

**Figure 3:**
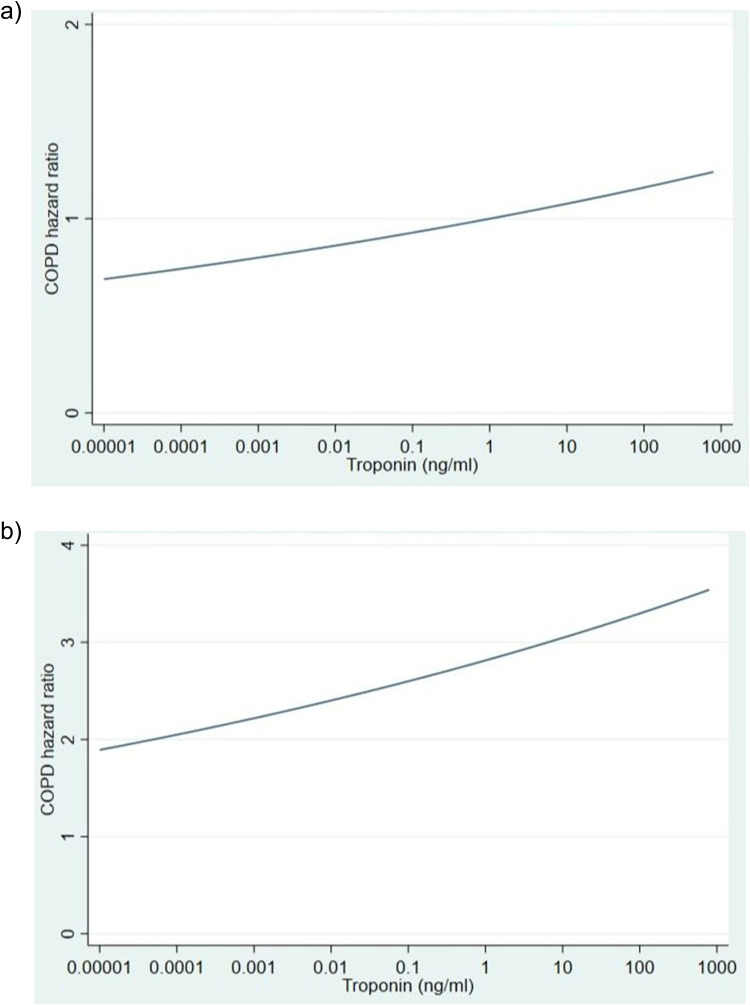
(**a**) Predicted unadjusted hazard ratio for COPD events (**b**) Predicted adjusted hazard ratio for COPD events.

We explored the relationship between troponin value and time to COPD readmission within 90 days. In this case, we found a significant linear effect for both unadjusted (HR 1.08, 95% CI 1.00–1.16, p = 0.043) and adjusted association (HR 1.08, 95% CI 1.00–1.17, p = 0.037). Harrell’s C coefficient and Gonen and Heller’s K coefficient were estimated to be equal to 0.60. We also investigated the relationship between troponin value and time to COPD readmission within 90 days for patients with elevated troponin (n = 1107) (Figure 3a and b).

#### Prediction of MACE Within 1 Year

**Figure 4:**
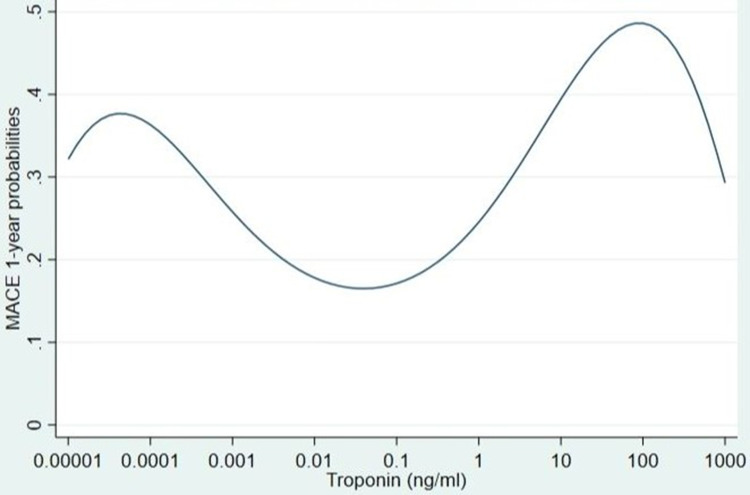
Predicted probabilities for 1-year MACE events.

We investigated the relationship between peak troponin value and having at least one MACE event within a year (n = 653, 26.3%). For each 10-fold increase of troponin, the effect increased and then decreased twice, below and above peak troponin threshold (Table 3 and Figure 4).

**Table 3:** Predicted Probabilities for 1-Year MACE Events

Model	Adjusted Predicted Probabilities for 1-Year MACE
Troponin (ng/mL)	P
0.00001	0.32
0.0001	0.36
0.001	0.26
0.01	0.18
0.1	0.17
1	0.25
10	0.39
100	0.49
1000	0.29

The Area under ROC curve for the model was 0.72 (95% CI 0.70–0.74). The AUC estimate is likely to be optimistic^17^ as the same observations are used to build this model and evaluate this model’s predictive performance. The LOOCV AUC was 0.71 (95% CI 0.68–0.73) and it was significantly lower when compared with the overall sample AUC (0.72) (p<0.001).

In addition, for patients with elevated troponin we investigated the relationship between troponin value and having at least one MACE event within a year (n = 410, 36.6%). We implemented logarithmic transformation (base-10 logarithm) in our analysis (log_10_ troponin) using a linear term (OR 1.65, 95% CI 1.26–2.16, p<0.001). Figure 4 illustrates the relationship between troponin and MACE probabilities. For each 10-fold increase of troponin, the effect increased. The Area under ROC curve for the model was 0.68 (95% CI 0.65–0.71). The AUC estimate is likely to be optimistic^17^ as the same observations are used to build this model and evaluate this model’s predictive performance. The LOOCV AUC was 0.65 (95% CI 0.62–0.69) and it was significantly lower when compared with the overall sample AUC (0.68) (p<0.001).

## Discussion

We found that nearly half of the cohort had elevated troponin when admitted with a COPD exacerbation. Further, 15% of our cohort had a MACE within 90 days from peak troponin measurement. Peak troponin was associated with an increased risk of MACE and COPD readmissions. Among those with an elevated troponin level, for each 10-fold increase of troponin, the effect increased and then decreased. When we used the data available to develop a prediction model for MACE within 90 days and MACE within 1 year, we found that the values for both coefficients were above the acceptable performance threshold but below a good performance threshold, suggesting that additional predictors would be required to improve predictive performance.

Troponin is commonly measured at the time of a COPD exacerbation, and several studies have reported elevated troponin levels at this time and shown associations with adverse outcomes, including rehospitalisation and increased mortality.^18–20^ It is still unclear, however, even with these data, whether investigation of the cause of the troponin rise would be beneficial in terms of altering clinical outcomes, including the likelihood of acute MACE events. Instead, it is noted that the cause of the elevated troponin in these populations is unclear, with studies suggesting that the elevated troponin may often be a result of systemic inflammation-induced myocardial injury.^5^,^21^,^22^ We are aware that the effect of troponin on time to MACE events within 90 days might be modified by interventions that might lead to better treatment and prevention of future MACE events. One intervention that we would have liked to have included as an effect modifier is coronary angiography but did not have sufficient power to do so. Furthermore, undertaking coronary angiography in patients with an admission with a COPD exacerbation is unlikely in the absence of ongoing classical ischaemic chest pain, regardless of the troponin level and very few individuals in our dataset underwent angiography. There are few readily available tests which front-line staff can use to determine the reason for the troponin elevation, although there are recent data to suggest that CT coronary angiography may be a promising tool for this purpose.^22^

The advent of hsTrop has precipitated considerable diagnostic uncertainty in routine clinical practice, for several reasons. The 99th centile level for assays, taken as the upper limit of normal (ULN) is derived from a few hundred relatively healthy individuals. However, 1 in 20 hospital patients (in- or out-patients) undergoing a blood test for any reason has a hsTrop above the ULN, even though in the vast majority there is no suspicion of a cardiac event.^6^ Further, the elevated hsTrop in a hospital population is most commonly due to myocardial injury or Type 2 myocardial infarction, rather than Type 1 MI, as defined by the Fourth Universal definition. An awareness of these data has increasingly led to the feeling that modest hsTrop elevations in patients such as those with COPD are unimportant unless they present with classical cardiac chest pain. However, there is an accumulating body of evidence suggesting that hsTrop elevation is always associated with an adverse outcome.^5^,^21^ For example, hsTrop levels in 20,000 consecutive hospital patients, most of whom had no suspicion of an acute CV event, was associated with 1-year mortality.^11^ Similarly, hsTrop levels were associated with outcomes in emergency room and intensive care patients.^23^,^24^

These previous findings are consistent with our study and indicate that there is potential clinical value in using hsTrop levels to predict outcome in patients admitted with exacerbation of COPD. However, such risk stratification would be of much greater value if future research could yield a pathway in which detection of elevated troponin then led to precise diagnosis and intervention that altered the subsequent event rate. The next step would be to conduct a trial in which troponin elevation is aggressively investigated in one arm using CTCA and stress MRI to detect potential avenues for interventional management for those detectable underlying conditions.

This study had several strengths, including the use of ICD-10 codes to define diseases. However, there are several important limitations in our work. The majority of MACE were due to all-cause mortality, we were not able to explore non-fatal or cardiovascular-specific mortality events. We cannot be sure that those people who had troponin measured at the time of exacerbation really are generalisable to all people hospitalised with a COPD exacerbation. Despite the database being considerably large and cross-regional, there were missing data for many of the variables. We were also not able to explore COPD-specific factors such as disease severity or medications in the analysis. We were also unable to adjust for a history of previous exacerbation episodes, which is a major predictor of further rehospitalisation.^3^ Angiography and PCI rates were perhaps lower than expected in our study. Information on Myocardial Perfusion (MIBI) scan, dobutamine stress echo and exercise stress tests (GXT) was not available and this may account for this finding. Information on drug treatments such as heparin drip, anticoagulation, antiplatelet therapy, statin use and beta-blocker use was not available and as such we were not able to explore treatment differences in people with COPD exacerbation with and without elevated troponin.

## Conclusions

An elevated troponin level at the time of COPD exacerbation may be a useful tool for predicting MACE events in patients with COPD. The relationship between degree of troponin elevation and risk of future events is complex and demands further investigation.
